# Supplementary figures and images for: How mindfulness, self-compassion, and experiential avoidance are related to perceived stress in a sample of university students
Source: PLoS One. 2023 Feb 3;18(2):e0280791. doi: 10.1371/journal.pone.0280791 (PMC9897529; doi:10.1371/journal.pone.0280791)

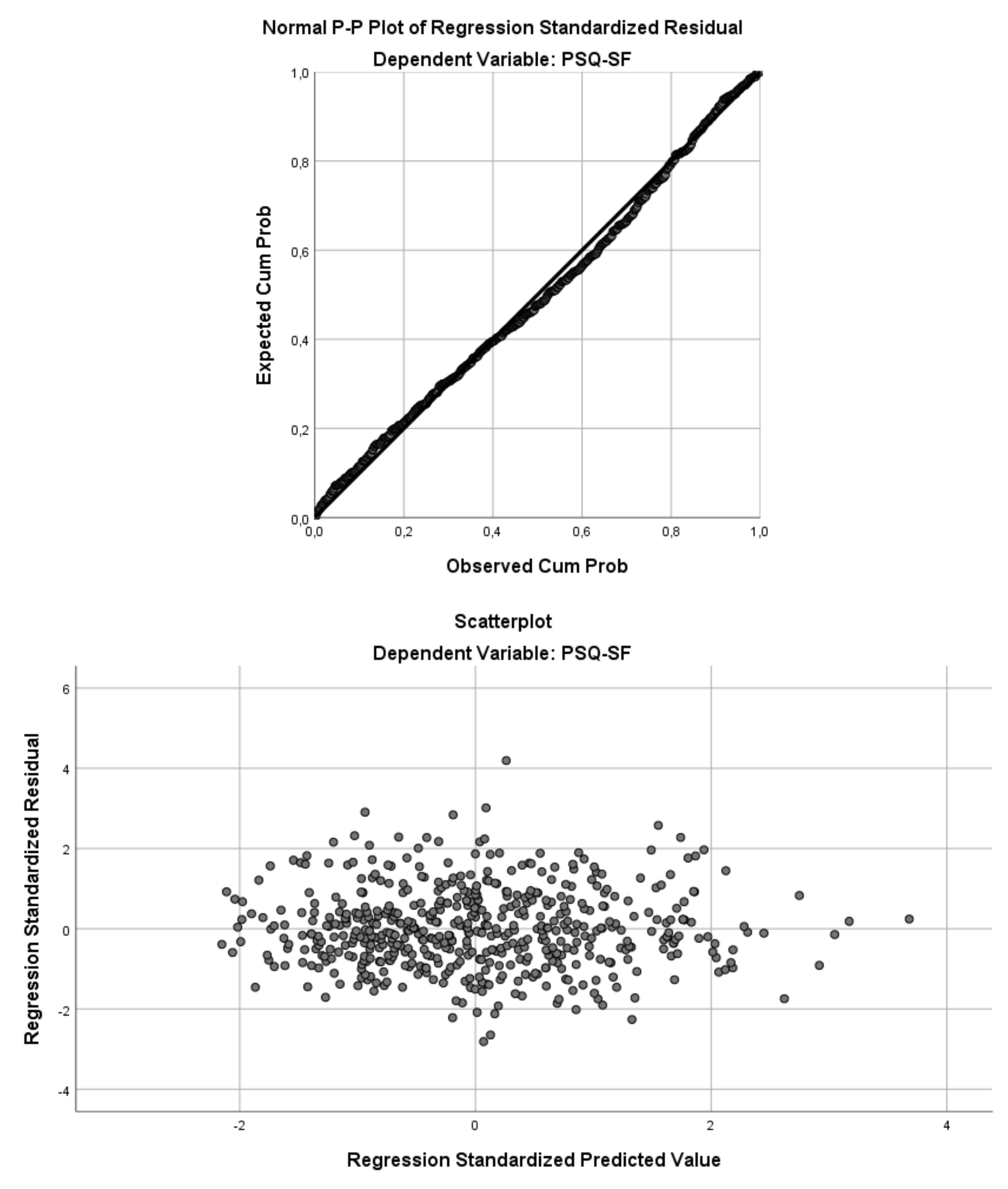

Supplement: S1 Fig — (TIF) [file pone.0280791.s001.tif]
