# Supplementary material for: How mindfulness, self-compassion, and experiential avoidance are related to perceived stress in a sample of university students
Source: PLoS One. 2023 Feb 3;18(2):e0280791. doi: 10.1371/journal.pone.0280791 (PMC9897529; doi:10.1371/journal.pone.0280791)
Supplement: S1 Table — (DOCX) [file pone.0280791.s002.docx]

| **Variable** | |  | **1** | | | **2** | | | **3** | | | **4** | | |  |  |
| --- | --- | --- | --- | --- | --- | --- | --- | --- | --- | --- | --- | --- | --- | --- | --- | --- |
| 1. PSQ |  | Pearson's r |  | — |  | |  |  | |  |  | |  |  | |  |
|  |  | p-value |  | — |  | |  |  | |  |  | |  |  | |  |
| 2. FFMQ |  | Pearson's r |  | -0.340 | *** | | — |  | |  |  | |  |  | |  |
|  |  | p-value |  | < .001 |  | | — |  | |  |  | |  |  | |  |
| 3. SCS |  | Pearson's r |  | -0.538 | *** | | 0.490 | *** | | — |  | |  |  | |  |
|  |  | p-value |  | < .001 |  | | < .001 |  | | — |  | |  |  | |  |
| 4. AAQ |  | Pearson's r |  | 0.611 | *** | | -0.482 | *** | | -0.633 | *** | | — |  | |  |
|  |  | p-value |  | < .001 |  | | < .001 |  | | < .001 |  | | — |  | |  |
| 5. Age |  | Pearson's r |  | 0.078 |  | | 0.129 | ** | | 0.150 | *** | | -0.066 |  | |  |
|  |  | p-value |  | 0.062 |  | | 0.002 |  | | < .001 |  | | 0.112 |  | |  |
| 6. Gender (0 = male; 1 = female) |  | Point-biserial correlation |  | 0.158 | *** | | -0.007 |  | | -0.180 | *** | | 0.098 | * | |  |
|  |  | p-value |  | < .001 |  | | 0.868 |  | | < .001 |  | | 0.018 |  | |  |
| 7. Having a partner (0 = no, 1 = yes) |  | Point-biserial correlation |  | -0.006 |  | | 0.076 |  | | 0.083 | * | | -0.212 | *** | |  |
|  |  | p-value |  | 0.888 |  | | 0.070 |  | | 0.044 |  | | < .001 |  | |  |
| 8. Children (0 = no, 1 = yes) |  | Point-biserial correlation |  | 0.063 |  | | 0.076 |  | | 0.067 |  | | 0.028 |  | |  |
|  |  | p-value |  | 0.125 |  | | 0.071 |  | | 0.103 |  | | 0.495 |  | |  |
| 9. Perceived family support (0 = no, 1 = yes) |  | Point-biserial correlation |  | -0.224 | *** | | -0.041 |  | | 0.006 |  | | -0.096 | * | |  |
|  |  | p-value |  | < .001 |  | | 0.334 |  | | 0.887 |  | | 0.019 |  | |  |
| 10. Employment (0 = no, 1 = yes) |  | Point-biserial correlation |  | 0.102 | * | | 0.045 |  | | -0.025 |  | | 0.012 |  | |  |
|  |  | p-value |  | 0.014 |  | | 0.287 |  | | 0.544 |  | | 0.775 |  | |  |
| 11. Left home (0 = no, 1 = yes) |  | Point-biserial correlation |  | 0.089 | * | | 0.005 |  | | 0.001 |  | | 0.017 |  | |  |
|  |  | p-value |  | 0.030 |  | | 0.903 |  | | 0.973 |  | | 0.681 |  | |  |
| 12. Scholarship (0 = no, 1 = yes) |  | Point-biserial correlation |  | < 0.001 |  | | 0.015 |  | | 0.020 |  | | 0.007 |  | |  |
|  |  | p-value |  | 0.983 |  | | 0.723 |  | | 0.623 |  | | 0.859 |  | |  |
| 13. Being at 1st academic year (0 = no, 1 = yes) |  | Point-biserial correlation |  | -0.072 |  | | -0.088* |  | | -0.014 |  | | 0.019 |  | |  |
|  |  | p-value |  | 0.079 |  | | 0.036 |  | | 0.742 |  | | 0.650 |  | |  |
| 14. Being at 2nd academic year (0 = no, 1 = yes) |  | Point-biserial correlation |  | 0.071 |  | | 0.034 |  | | -0.029 |  | | 0.059 |  | |  |
|  |  | p-value |  | 0.087 |  | | 0.412 |  | | 0.489 |  | | 0.151 |  | |  |
| 15. Being at 3rd academic year (0 = no, 1 = yes) |  | Point-biserial correlation |  | 0.040 |  | | 0.021 |  | | -0.044 |  | | 0.041 |  | |  |
|  |  | p-value |  | 0.333 |  | | 0.625 |  | | 0.286 |  | | 0.323 |  | |  |
| 16. Being at 4th academic year (0 = no, 1 = yes) |  | Point-biserial correlation |  | -0.049 |  | | 0.052 |  | | 0.098* |  | | -0.080 |  | |  |
|  |  | p-value |  | 0.235 |  | | 0.213 |  | | 0.018 |  | | 0.052 |  | |  |
| 17. Being at 5th academic year (0 = no, 1 = yes) |  | Point-biserial correlation |  | 0.016 |  | | -0.040 |  | | -0.028 |  | | -0.055 |  | |  |
|  |  | p-value |  | 0.702 |  | | 0.335 |  | | 0.491 |  | | 0.183 |  | |  |
| 18. Study hours per week |  | Pearson's r |  | 0.158 | *** | | 0.138 | *** | | -0.048 |  | | 0.014 |  | |  |
|  |  | p-value |  | < .001 |  | | < .001 |  | | 0.243 |  | | 0.737 |  | |  |
| 19. Number of failed subjects |  | Pearson's r |  | 0.084* |  | | 0.033 |  | | -0.036 |  | | 0.023 |  | |  |
|  |  | p-value |  | 0.042 |  | | 0.439 |  | | 0.386 |  | | 0.576 |  | |  |

*Note:* Values are Pearson’s r (for continuous variables with continuous variable) or point-biserial (for continuous variable with dichotomous variables) correlations. AAQ = Acceptance and Action Questionnaire-II; FFMQ = Five Facets of Mindfulness Questionnaire-Short Form; PSQ = Perceived Stress Questionnaire-24-item version; SCS = Self-Compassion Scale-Short Form.

* *p* < .05; ** *p* < .01; *** *p* <.001
