# Supplementary material for: How mindfulness, self-compassion, and experiential avoidance are related to perceived stress in a sample of university students
Source: PLoS One. 2023 Feb 3;18(2):e0280791. doi: 10.1371/journal.pone.0280791 (PMC9897529; doi:10.1371/journal.pone.0280791)
Supplement: S2 Table — (DOCX) [file pone.0280791.s003.docx]

| **Coefficients^a^** | | | | | | | | | | | | | |
| --- | --- | --- | --- | --- | --- | --- | --- | --- | --- | --- | --- | --- | --- |
| Model | | Unstandarized Coefficients | | Standardized Coefficients | t | Sig. | 95% CI B | | Correlations | | | Colinearity statistics | |
|  |  | B | Std. Error | Beta |  |  | Lower limit | Upper limit | Zero-order | Partial | Part | Tolerance | VIF |
| 1 | (Constant) | .400 | .078 |  | 5.108 | .000 | .246 | .554 |  |  |  |  |  |
|  | Age | .001 | .002 | .033 | .638 | .524 | -.003 | .006 | .078 | .027 | .026 | .613 | 1.630 |
|  | Gender (0 = male; 1 = female) | .070 | .018 | .160 | 3.852 | .000 | .034 | .106 | .158 | .163 | .155 | .938 | 1.066 |
|  | Having a partner (0 = no. 1 = yes) | -.006 | .014 | -.016 | -.394 | .694 | -.034 | .022 | -.006 | -.017 | -.016 | .948 | 1.055 |
|  | Children (0 = no. 1 = yes) | .009 | .058 | .007 | .152 | .879 | -.105 | .123 | .063 | .007 | .006 | .848 | 1.179 |
|  | Perceived family support (0 = no. 1 = yes) | -.182 | .035 | -.221 | -5.218 | .000 | -.250 | -.113 | -.224 | -.218 | -.209 | .900 | 1.111 |
|  | Employment (0 = no. 1 = yes) | .035 | .019 | .078 | 1.905 | .057 | -.001 | .072 | .102 | .081 | .076 | .960 | 1.041 |
|  | Left home (0 = no. 1 = yes) | .038 | .019 | .087 | 2.024 | .043 | .001 | .075 | .089 | .086 | .081 | .880 | 1.136 |
|  | Scholarship (0 = no. 1 = yes) | .004 | .016 | .009 | .221 | .826 | -.028 | .035 | .001 | .009 | .009 | .939 | 1.065 |
|  | Being at 1st academic year (0 = no. 1 = yes) | .001 | .022 | .002 | .046 | .964 | -.043 | .045 | -.072 | .002 | .002 | .598 | 1.671 |
|  | Being at 2nd academic year (0 = no. 1 = yes) | .037 | .021 | .092 | 1.748 | .081 | -.005 | .078 | .071 | .075 | .070 | .583 | 1.716 |
|  | Being at 3rd academic year (0 = no. 1 = yes) | .020 | .021 | .046 | .931 | .352 | -.022 | .061 | .040 | .040 | .037 | .660 | 1.515 |
|  | Being at 5th academic year (0 = no. 1 = yes) | .009 | .029 | .014 | .313 | .755 | -.048 | .067 | .016 | .013 | .013 | .823 | 1.215 |
|  | Study hours per week | .002 | .001 | .122 | 2.915 | .004 | .001 | .003 | .158 | .124 | .117 | .915 | 1.093 |
|  | Number of failed subjects | .001 | .003 | .018 | .417 | .677 | -.005 | .008 | .084 | .018 | .017 | .867 | 1.154 |
| 2 | (Constant) | .386 | .082 |  | 4.715 | .000 | .225 | .547 |  |  |  |  |  |
|  | Age | .004 | .002 | .095 | 2.433 | .015 | .001 | .008 | .078 | .104 | .074 | .604 | 1.657 |
|  | Gender (0 = male; 1 = female) | .036 | .014 | .082 | 2.579 | .010 | .009 | .064 | .158 | .110 | .078 | .909 | 1.100 |
|  | Having a partner (0 = no. 1 = yes) | .031 | .011 | .091 | 2.858 | .004 | .010 | .053 | -.006 | .122 | .087 | .902 | 1.109 |
|  | Children (0 = no. 1 = yes) | -.001 | .044 | -.001 | -.018 | .986 | -.087 | .086 | .063 | -.001 | -.001 | .843 | 1.186 |
|  | Perceived family support (0 = no. 1 = yes) | -.137 | .027 | -.167 | -5.179 | .000 | -.190 | -.085 | -.224 | -.217 | -.157 | .885 | 1.130 |
|  | Employment (0 = no. 1 = yes) | .027 | .014 | .059 | 1.902 | .058 | -.001 | .054 | .102 | .081 | .058 | .955 | 1.047 |
|  | Left home (0 = no. 1 = yes) | .022 | .014 | .050 | 1.540 | .124 | -.006 | .050 | .089 | .066 | .047 | .877 | 1.140 |
|  | Scholarship (0 = no. 1 = yes) | .002 | .012 | .006 | .207 | .836 | -.021 | .026 | .001 | .009 | .006 | .938 | 1.066 |
|  | Being at 1st academic year (0 = no. 1 = yes) | -.007 | .017 | -.017 | -.444 | .657 | -.040 | .025 | -.072 | -.019 | -.013 | .598 | 1.673 |
|  | Being at 2nd academic year (0 = no. 1 = yes) | .026 | .016 | .066 | 1.661 | .097 | -.005 | .058 | .071 | .071 | .050 | .580 | 1.723 |
|  | Being at 3rd academic year (0 = no. 1 = yes) | .002 | .016 | .006 | .157 | .876 | -.029 | .034 | .040 | .007 | .005 | .657 | 1.523 |
|  | Being at 5th academic year (0 = no. 1 = yes) | .005 | .022 | .008 | .244 | .808 | -.038 | .049 | .016 | .010 | .007 | .815 | 1.227 |
|  | Study hours per week | .002 | .001 | .125 | 3.898 | .000 | .001 | .003 | .158 | .165 | .118 | .891 | 1.122 |
|  | Number of failed subjects | .001 | .002 | .019 | .580 | .562 | -.003 | .006 | .084 | .025 | .018 | .866 | 1.154 |
|  | FFMQ-SF | -.001 | .001 | -.072 | -1.940 | .053 | -.003 | .000 | -.340 | -.083 | -.059 | .662 | 1.511 |
|  | SCS-SF | -.005 | .001 | -.229 | -5.453 | .000 | -.006 | -.003 | -.538 | -.228 | -.165 | .520 | 1.923 |
|  | AAQ-II | .008 | .001 | .426 | 10.066 | .000 | .006 | .010 | .611 | .397 | .305 | .512 | 1.952 |
| a. Dependent variable: PSQ-SF. FFMQ-SF = Five Facets of Mindfulness Questionnaire-Short Form; SCS-SF = Self-Compassion Scale-Short Form; AAQ-II = Acceptance and Action Questionnaire-II; PSQ-SF = Perceived Stress Questionnaire-24-item version. | | | | | | | | | | | | | |
