# Supplementary material for: How mindfulness, self-compassion, and experiential avoidance are related to perceived stress in a sample of university students
Source: PLoS One. 2023 Feb 3;18(2):e0280791. doi: 10.1371/journal.pone.0280791 (PMC9897529; doi:10.1371/journal.pone.0280791)
Supplement: S3 Table — (DOCX) [file pone.0280791.s004.docx]

| **Model summary** | | | | | | | | | |
| --- | --- | --- | --- | --- | --- | --- | --- | --- | --- |
| Model | R | R square | Adjusted R Square | Std. Error of the Estimate | Change Statistics | | | | |
|  |  |  |  |  | R Square Change | F Change | df1 | df2 | Sig. F Change |
| 1 | .350^a^ | .123 | .100 | .16367 | .123 | 5.440 | 14 | 545 | .000 |
| 2 | .703^b^ | .494 | .478 | .12460 | .372 | 132.775 | 3 | 542 | .000 |

| **ANOVA** | | | | | | |
| --- | --- | --- | --- | --- | --- | --- |
| Model | | Sum of Squares | df | Mean Square | F | Sig. |
| 1 | Regression | 2.040 | 14 | .146 | 5.440 | .000^b^ |
|  | Residual | 14.600 | 545 | .027 |  |  |
|  | Total | 16.640 | 559 |  |  |  |
| 2 | Regression | 8.225 | 17 | .484 | 31.160 | .000^c^ |
|  | Residual | 8.415 | 542 | .016 |  |  |
|  | Total | 16.640 | 559 |  |  |  |

| **Coefficients^a^** | | | | | | | | | | | | | |
| --- | --- | --- | --- | --- | --- | --- | --- | --- | --- | --- | --- | --- | --- |
| Model | | Unstandardized coefficients | | Standardized coefficients | t | Sig. | 95% CI B | | Correlations | | | Colinearity statistics | |
|  |  | B | Std. Error | Beta |  |  | Lower limit | Upper limit | Zero-order | Partial | Part | Tolerance | VIF |
| 1 | (Constant) | .400 | .078 |  | 5.108 | .000 | .246 | .554 |  |  |  |  |  |
|  | Age | .001 | .002 | .033 | .638 | .524 | -.003 | .006 | .078 | .027 | .026 | .613 | 1.630 |
|  | Gender (0 = male; 1 = female) | .070 | .018 | .160 | 3.852 | .000 | .034 | .106 | .158 | .163 | .155 | .938 | 1.066 |
|  | Having a partner (0 = no. 1 = yes) | -.006 | .014 | -.016 | -.394 | .694 | -.034 | .022 | -.006 | -.017 | -.016 | .948 | 1.055 |
|  | Children (0 = no. 1 = yes) | .009 | .058 | .007 | .152 | .879 | -.105 | .123 | .063 | .007 | .006 | .848 | 1.179 |
|  | Perceived family support (0 = no. 1 = yes) | -.182 | .035 | -.221 | -5.218 | .000 | -.250 | -.113 | -.224 | -.218 | -.209 | .900 | 1.111 |
|  | Employment (0 = no. 1 = yes) | .035 | .019 | .078 | 1.905 | .057 | -.001 | .072 | .102 | .081 | .076 | .960 | 1.041 |
|  | Left home (0 = no. 1 = yes) | .038 | .019 | .087 | 2.024 | .043 | .001 | .075 | .089 | .086 | .081 | .880 | 1.136 |
|  | Scholarship (0 = no. 1 = yes) | .004 | .016 | .009 | .221 | .826 | -.028 | .035 | .001 | .009 | .009 | .939 | 1.065 |
|  | Being at 1st academic year (0 = no. 1 = yes) | .001 | .022 | .002 | .046 | .964 | -.043 | .045 | -.072 | .002 | .002 | .598 | 1.671 |
|  | Being at 2nd academic year (0 = no. 1 = yes) | .037 | .021 | .092 | 1.748 | .081 | -.005 | .078 | .071 | .075 | .070 | .583 | 1.716 |
|  | Being at 3rd academic year (0 = no. 1 = yes) | .020 | .021 | .046 | .931 | .352 | -.022 | .061 | .040 | .040 | .037 | .660 | 1.515 |
|  | Being at 5th academic year (0 = no. 1 = yes) | .009 | .029 | .014 | .313 | .755 | -.048 | .067 | .016 | .013 | .013 | .823 | 1.215 |
|  | Study hours per week | .002 | .001 | .122 | 2.915 | .004 | .001 | .003 | .158 | .124 | .117 | .915 | 1.093 |
|  | Number of failed subjects | .001 | .003 | .018 | .417 | .677 | -.005 | .008 | .084 | .018 | .017 | .867 | 1.154 |
| 2 | (Constant) | .357 | .083 |  | 4.319 | .000 | .195 | .520 |  |  |  |  |  |
|  | Age | .004 | .002 | .086 | 2.200 | .028 | .000 | .007 | .078 | .094 | .067 | .606 | 1.650 |
|  | Gender (0 = male; 1 = female) | .042 | .014 | .095 | 2.977 | .003 | .014 | .069 | .158 | .127 | .091 | .921 | 1.086 |
|  | Having a partner (0 = no. 1 = yes) | .034 | .011 | .098 | 3.067 | .002 | .012 | .056 | -.006 | .131 | .094 | .905 | 1.105 |
|  | Children (0 = no. 1 = yes) | .006 | .044 | .005 | .143 | .887 | -.081 | .094 | .063 | .006 | .004 | .838 | 1.194 |
|  | Perceived family support (0 = no. 1 = yes) | -.135 | .027 | -.164 | -5.051 | .000 | -.188 | -.083 | -.224 | -.212 | -.154 | .885 | 1.130 |
|  | Employment (0 = no. 1 = yes) | .025 | .014 | .056 | 1.790 | .074 | -.002 | .053 | .102 | .077 | .055 | .951 | 1.052 |
|  | Left home (0 = no. 1 = yes) | .022 | .014 | .049 | 1.510 | .132 | -.007 | .050 | .089 | .065 | .046 | .877 | 1.140 |
|  | Scholarship (0 = no. 1 = yes) | .003 | .012 | .009 | .277 | .782 | -.021 | .027 | .001 | .012 | .008 | .936 | 1.068 |
|  | Being at 1st academic year (0 = no. 1 = yes) | -.008 | .017 | -.018 | -.455 | .649 | -.041 | .026 | -.072 | -.020 | -.014 | .598 | 1.673 |
|  | Being at 2nd academic year (0 = no. 1 = yes) | .026 | .016 | .064 | 1.602 | .110 | -.006 | .057 | .071 | .069 | .049 | .580 | 1.724 |
|  | Being at 3rd academic year (0 = no. 1 = yes) | .004 | .016 | .010 | .258 | .797 | -.027 | .036 | .040 | .011 | .008 | .657 | 1.521 |
|  | Being at 5th academic year (0 = no. 1 = yes) | .004 | .022 | .007 | .194 | .846 | -.040 | .048 | .016 | .008 | .006 | .813 | 1.230 |
|  | Study hours per week | .002 | .001 | .130 | 4.029 | .000 | .001 | .003 | .158 | .171 | .123 | .894 | 1.119 |
|  | Number of failed subjects | .001 | .002 | .018 | .553 | .580 | -.003 | .006 | .084 | .024 | .017 | .866 | 1.155 |
|  | FFMQ-SF | -.002 | .001 | -.090 | -2.430 | .015 | -.003 | .000 | -.340 | -.104 | -.074 | .678 | 1.475 |
|  | Ad hoc SCS-12 | -.006 | .001 | -.179 | -4.432 | .000 | -.008 | -.003 | -.501 | -.187 | -.135 | .569 | 1.756 |
|  | AAQ-II | .009 | .001 | .453 | 10.800 | .000 | .007 | .010 | .611 | .421 | .330 | .530 | 1.888 |
| ^a^ Dependent variable: PSQ-SF. FFMQ-SF = Five Facets of Mindfulness Questionnaire-Short Form; Ad hoc SCS-SF = Self-Compassion Scale-Short Form (without mindfulness and overidentification items); AAQ-II = Acceptance and Action Questionnaire-II; PSQ-SF = Perceived Stress Questionnaire-24-item version. Variable ‘Being at 4th academic year’ was excluded by SPSS from the models because showed impossible tolerance values. | | | | | | | | | | | | | |
