# Supplementary material for: How mindfulness, self-compassion, and experiential avoidance are related to perceived stress in a sample of university students
Source: PLoS One. 2023 Feb 3;18(2):e0280791. doi: 10.1371/journal.pone.0280791 (PMC9897529; doi:10.1371/journal.pone.0280791)
Supplement: Attachment — Submitted filename: PONE-D-21-35822.docx [file pone.0280791.s005.docx]

**How Experiential Avoidance, Self-Compassion, and Mindfulness are related to Perceived Stress in a Sample of University Students of Education**

This study explored how mindfulness, self-compassion, and experiential avoidance were related to stress in university students. While this is an important area of research, I have concerns about several areas of the manuscript, which I have outlined below. In its current form, I do not believe that this manuscript can meaningfully contribute to the literature and recommend a major revision.

**Introduction**

- Overall, the introduction does not tell a coherent story to work towards setting up the current study aims. There are related ideas spread across several paragraphs that would benefit from being integrated together. Other paragraphs do not seem necessary (e.g., that teachers experience stress), but rather distract from the study aims. Most importantly, the key focus of the study is around how mindfulness, self-compassion, and experiential avoidance relate to stress, but this is lacking within the introduction. I suggest that the authors identify the 1-2 main aims of their paper and reorganize the introduction section accordingly to better help with flow and interpretability.

Other suggestions:

- The authors should include a first, initial paragraph that introduces the topic and the study aims for their paper, before summarizing the literature.
- The focus of the paper is on dispositional mindfulness, but the introduction only briefly mentions how mindfulness trainings are related to stress. The connection between dispositional mindfulness and stress, especially among college students, needs to be described to better set up the paper.
- Self-compassion is suggested as a potential mediator of the relationship between mindfulness and happiness. By bringing this up, I wondered if the authors would test this mediation with stress as an outcome. The focus of the paragraph should be about the association between self-compassion and stress among college students.
- Experiential avoidance is discussed as a potential moderator of several associations, including anxiety, addictions, etc. Again, the authors need to discuss literature about how it is related to stress in particular to better set up the study aims.
- It will also be beneficial to discuss how mindfulness, self-compassion, and experiential avoidance are associated with each other.
- The study aims should be reframed to focus on exploring associations among mindfulness, self-compassion, and experiential avoidance with stress. Do the authors have any hypotheses?

**Methods**

- Additional descriptions of study measures should be included to better interpret some of the measures. How was presence of family support measured (yes/no; good/bad)? How were overall scores created (summed, averaged)?
- Why did the authors choose to explore the five facets of mindfulness instead of an overall index score? This rationale should be included. The non-reactivity subscale demonstrated low reliability, which should be noted as a limitation. Was a Spanish version of the mindfulness scale used? If so, this should be stated.

**Procedure**

- What were the students informed that the study was about?
- What year was the study conducted in?
- Were the students compensated for participating?
- Were there any differences between students who completed the survey online vs. those who completed the survey using a pencil and paper?

**Results**

- How were missing data handled?
- The authors stated that stress and other variables corresponded to “a medium score range.” What exactly do the authors mean by this? Are there cut-offs for clinically meaningful levels of stress? Are there references for interpreting what these values mean?
- The paragraph describing the description of the sample repeats all of the info in Table 1. Unless the authors want to describe clinically meaningful cut-off scores based on the means of the variables, I think this information can be removed from the text and only included in the table.
- I suggest that the authors do not use a stepwise regression for their analyses. A stronger approach would be a hierarchical multiple regression based on variables associated with stress at the univariable level (correlations). This would still examine whether mindfulness, self-compassion, and experiential avoidance are associated with stress above the effects of sociodemographic and academic variables.

**Tables**

- Table 1
  - The authors should include a note under the table explaining what the abbreviations for the measures correspond to.
  - Please add the range of scores for each measure within your sample.
- Table 2
  - This table is very large and difficult to read.
  - The key study variables should be listed at the top of the table (stress, mindfulness, self-compassion, and experiential avoidance).
  - The authors should include a note under the table explaining how variables were dichotomized. For example, what does a negative vs. positive correlation for gender mean?

**Discussion**

- The first paragraph does not highlight the main findings from the regression.
- Some facets of mindfulness were not associated in the direction as other facets. Why do the authors think that is?
- Why do the authors think that the acting with awareness facet in particular was important, relative to the other facets? What are the implications of these findings?
- The authors mention how self-compassion overlaps with mindfulness. Indeed, self-compassion includes a component of mindfulness. What are the potential methodological concerns with using both constructs in the regression model? Did the authors test self-compassion with the mindfulness component removed?
- Having an in-person, pencil and paper survey could cause potential bias in responses. This should be discussed.

**Other**

- Sometimes abbreviations for stress “PSQ” are used and other times it is referred to as “stress”. Be more consistent throughout the text to reduce confusion.
- Sometimes the variables are referred to as “academic” variables and other time “occupational” variables (table titles; section headings). Be more consistent throughout the text to reduce confusion.
- There are several grammatical and spelling errors.
- Have the data from this study been reported elsewhere?
