# Supplementary material for: How mindfulness, self-compassion, and experiential avoidance are related to perceived stress in a sample of university students
Source: PLoS One. 2023 Feb 3;18(2):e0280791. doi: 10.1371/journal.pone.0280791 (PMC9897529; doi:10.1371/journal.pone.0280791)
Supplement: Attachment — Submitted filename: Response to reviewers.docx [file pone.0280791.s006.docx]

***Journal requirements***

When submitting your revision, we need you to address these additional requirements.

1. Please ensure that your manuscript meets PLOS ONE's style requirements, including those for file naming. The PLOS ONE style templates can be found at

<https://journals.plos.org/plosone/s/file?id=wjVg/PLOSOne_formatting_sample_main_body.pdf>

and

<https://journals.plos.org/plosone/s/file?id=ba62/PLOSOne_formatting_sample_title_authors_affiliations.pdf>.

**Answer:** The paper has been formatted according to PLOS ONE’s style requirements.

2. Please provide additional details regarding participant consent. In the ethics statement in the Methods and online submission information, please ensure that you have specified (1) whether consent was informed and (2) what type you obtained (for instance, written or verbal, and if verbal, how it was documented and witnessed). If your study included minors, state whether you obtained consent from parents or guardians. If the need for consent was waived by the ethics committee, please include this information.

If you are reporting a retrospective study of medical records or archived samples, please ensure that you have discussed whether all data were fully anonymized before you accessed them and/or whether the IRB or ethics committee waived the requirement for informed consent. If patients provided informed written consent to have data from their medical records used in research, please include this information.

**Answer:** We have specified the information about the consent (page 10): *“Students were invited to participate at the end of their classes. They were informed about the voluntary nature of participation and the confidentiality of the data. Concretely, it was explained to them that the aim of the study was to investigate the association among their levels of perceived stress, mindfulness, self-compassion, and experiential avoidance.* ***After that, we obtained written informed consent from all students interested in the study.*** *Then, they were asked to answer individually the study measures through a paper-and-pencil survey or via online format (Survey Monkey; 46% of the cases).”*

3. Peer review at PLOS ONE is not double-blinded (https://journals.plos.org/plosone/s/editorial-and-peer-review-process). For this reason, authors should include in the revised manuscript all the information removed for blind review.

**Answer:** Fixed. We have unblinded the manuscript.

4. Thank you for stating the following in the Acknowledgments Section of your manuscript:

“AC-C has a FI predoctoral contract from AGAUR (FI_B/00216). JPS-M has a PFIS predoctoral contract from the ISCIII (FI20/00034). AP-A has a Sara Borrell postdoctoral contract from the ISCIII (CD20/00181). JVL had a “Miguel Servet” research contract from the ISCIII (CPII19/00003) when the study was conducted. JM-M is supported by the Wellcome Trust Grant (104908/Z/14/Z/). AF-S and JVL acknowledge the funding from the Serra Húnter program (UAB-LE-8015 and UAB-LE-120014, respectively). The funding bodies did not play any role in the analysis and interpretation of data, in the writing of the manuscript, or in the decision to submit the paper for publication.”

We note that you have provided funding information that is not currently declared in your Funding Statement. However, funding information should not appear in the Acknowledgments section or other areas of your manuscript. We will only publish funding information present in the Funding Statement section of the online submission form.

Please remove any funding-related text from the manuscript and let us know how you would like to update your Funding Statement. Currently, your Funding Statement reads as follows:

“AC-C has a FI predoctoral contract from AGAUR (FI_B/00216). JPS-M has a PFIS predoctoral contract from the ISCIII (FI20/00034). AP-A has a Sara Borrell postdoctoral contract from the ISCIII (CD20/00181). JVL had a “Miguel Servet” research contract from the ISCIII (CPII19/00003) when the study was conducted. JM-M is supported by the Wellcome Trust Grant (104908/Z/14/Z/). AF-S and JVL acknowledge the funding from the Serra Húnter program (UAB-LE-8015 and UAB-LE-120014, respectively). The funding bodies did not play any role in the analysis and interpretation of data, in the writing of the manuscript, or in the decision to submit the paper for publication.”

Please include your amended statements within your cover letter; we will change the online submission form on your behalf.

**Answer:** Funding-related information has been removed from the paper. I confirm that the Funding Statement should read as follows:

AC-C has a FI predoctoral contract from AGAUR (FI_B/00216). JPS-M has a PFIS predoctoral contract from the ISCIII (FI20/00034). AP-A has a Sara Borrell postdoctoral contract from the ISCIII (CD20/00181). JN has a research contract from the Institute of Health Carlos III (ISCIII; ICI20/00080). JM-M has a “Miguel Servet” contract from the ISCIII (CP21/00080). JM-M was supported by the Wellcome Trust Grant (104908/Z/14/Z/) when the study was conducted. AF-S acknowledges the funding from the Serra Húnter program (Generalitat de Catalunya; reference number UAB-LE-8015). The funding bodies did not play any role in the analysis and interpretation of data, in the writing of the manuscript, or in the decision to submit the paper for publication.

5. Thank you for stating the following financial disclosure:

“AC-C has a FI predoctoral contract from AGAUR (FI_B/00216). JPS-M has a PFIS predoctoral contract from the ISCIII (FI20/00034). AP-A has a Sara Borrell postdoctoral contract from the ISCIII (CD20/00181). JVL had a “Miguel Servet” research contract from the ISCIII (CPII19/00003) when the study was conducted. JM-M is supported by the Wellcome Trust Grant (104908/Z/14/Z/). AF-S and JVL acknowledge the funding from the Serra Húnter program (UAB-LE-8015 and UAB-LE-120014, respectively). The funding bodies did not play any role in the analysis and interpretation of data, in the writing of the manuscript, or in the decision to submit the paper for publication.”

Please state what role the funders took in the study. If the funders had no role, please state: ""The funders had no role in study design, data collection and analysis, decision to publish, or preparation of the manuscript.""

If this statement is not correct you must amend it as needed.

Please include this amended Role of Funder statement in your cover letter; we will change the online submission form on your behalf.

**Answer:** I confirm that **‘**the funders had no role in study design, data collection and analysis, decision to publish, or preparation of the manuscript’.

6. In your Data Availability statement, you have not specified where the minimal data set underlying the results described in your manuscript can be found. PLOS defines a study's minimal data set as the underlying data used to reach the conclusions drawn in the manuscript and any additional data required to replicate the reported study findings in their entirety. All PLOS journals require that the minimal data set be made fully available. For more information about our data policy, please see http://journals.plos.org/plosone/s/data-availability.

Upon re-submitting your revised manuscript, please upload your study’s minimal underlying data set as either Supporting Information files or to a stable, public repository and include the relevant URLs, DOIs, or accession numbers within your revised cover letter. For a list of acceptable repositories, please see http://journals.plos.org/plosone/s/data-availability#loc-recommended-repositories. Any potentially identifying patient information must be fully anonymized.

Important: If there are ethical or legal restrictions to sharing your data publicly, please explain these restrictions in detail. Please see our guidelines for more information on what we consider unacceptable restrictions to publicly sharing data: http://journals.plos.org/plosone/s/data-availability#loc-unacceptable-data-access-restrictions. Note that it is not acceptable for the authors to be the sole named individuals responsible for ensuring data access.

We will update your Data Availability statement to reflect the information you provide in your cover letter.

**Answer:** Following your instructions, we have provided a link to OSF in which the data file associated to the present analysis can be downloaded:

“For transparency and analytical reproducibility purposes, SPSS data can be accessed at OSF: https://osf.io/g2sfp/”

**Reviewer #1**

**Authors**: We would like to thank you very much for your kind and thoughtful comments. We will provide an answer after each comment below.

Introduction

Overall, the introduction does not tell a coherent story to work towards setting up the current study aims. There are related ideas spread across several paragraphs that would benefit from being integrated together. Other paragraphs do not seem necessary (e.g., that teachers experience stress), but rather distract from the study aims. Most importantly, the key focus of the study is around how mindfulness, self-compassion, and experiential avoidance relate to stress, but this is lacking within the introduction. I suggest that the authors identify the 1-2 main aims of their paper and reorganize the introduction section accordingly to better help with flow and interpretability.

**Answer:** Following reviewer’s suggestion, we have entirely rewritten the introduction section following all your considerations. Changes can be checked throughout the whole section. Find below some paragraphs with major changes that address your concerns (pages 2-6):

*“The mental health of university students has become a worldwide concern [1]. It is estimated that around 35% of university students meet the diagnostic criteria for a mental disorder [2]. Furthermore, the pooled prevalence of depression among undergraduates is 25% and 14% for the prevalence of suicide-related outcomes [3]. In fact, their risk of developing a mental illness has significantly increased up to around 50% as a result of the COVID-19 pandemic [4,5]. Overall, university students are particularly vulnerable to psychological distress, which in this population is strongly related to academic underperformance, i.e. failure to fulfil academic obligations, and problematic health behaviours, such as substance use [6,7].*

*Given the high prevalence of mental disorders among university students, there has been a burgeoning interest in detecting potential protective skills that could be enhanced in order to improve their mental health, such as mindfulness, self-compassion, and psychological flexibility [8, 9]. These are three psychological constructs of “third wave” cognitive behavioural therapy, in which the focus is on the relationship between the individual and his/her thoughts and emotions [10].”*

*[…]*

*“Many studies have consistently indicated the negative association of mindfulness, compassion, and psychological flexibility with psychological distress in general populations [14,15]. Specifically in university students, for instance, Burger et al. [16] investigated the associations between psychological distress and mindfulness in a sample of 174 university students and found that higher levels of mindfulness (especially the facet of acting with awareness) significantly predicted lower levels of psychological distress.”*

*[…]*

*“Regarding compassion, Chan et al. [19] have found in a sample of 536 university students that compassion for others and self-compassion were linked to lower levels of psychological distress.”*

*[…]*

*“Regarding psychological flexibility, previous studies have focused on experiential avoidance, which is a process aimed at trying to facilitate psychological flexibility. Experiential avoidance is defined as a “phenomenon that occurs when a person is unwilling to remain in contact with particular private experiences (e.g., bodily sensations, emotions, thoughts, memories, behavioural predispositions) and takes steps to alter the form or frequency of these events and the contexts that occasion them” ([22], p. 1154).”*

*[…]*

*“Mindfulness, self-compassion, and experiential avoidance are three interrelated processes. According to Neff [27], there is a reciprocal association between mindfulness and self-compassion, in which each one is necessary for facilitating and enhancing the other.”*

*[…]*

*“Finally, previous studies on the association between sociodemographic variables and distress in university students have shown that, for example, gender plays an important role in stress symptomatology since being female is a vulnerability factor for developing it [29,30].”*

*[…]*

*“Overall, all these studies conclude that interventions aimed at increasing mindfulness facets, compassion, or psychological flexibility (by reducing experiential avoidance) may help to improve psychological distress in university students (e.g. [16,18,23]). However, very few studies have simultaneously studied the ability of trait mindfulness, compassion, and experiential avoidance, along with sociodemographic and academic variables, to explain levels of psychological distress in university students. Including all those variables in the same model would add specific information about which of these psychological constructs should be more emphasised in mental health interventions for university students.”*

*[…]*

*“Therefore, the aim of the current study was to explore the relative contributions of three processes that are therapeutically relevant to perceived stress (i.e. trait mindfulness, self-compassion, and experiential avoidance), a critical construct among university students who are at risk of burnout, depression, and anxiety.”*

*[…]*

Other suggestions:

The authors should include a first, initial paragraph that introduces the topic and the study aims for their paper, before summarizing the literature.

**Answer:** Following reviewer’s suggestion, we have begun the introduction section speaking about the topic of the study, i.e., the mental health status of university studies and the interest in detecting potential protective skills that could be enhanced to improve their mental health (page 2):

*“The mental health of university students has become a worldwide concern [1]. It is estimated that around 35% of university students meet the diagnostic criteria for a mental disorder [2]. Furthermore, the pooled prevalence of depression among undergraduates is 25% and 14% for the prevalence of suicide-related outcomes [3]. In fact, their risk of developing a mental illness has significantly increased up to around 50% as a result of the COVID-19 pandemic [4,5]. Overall, university students are particularly vulnerable to psychological distress, which in this population is strongly related to academic underperformance, i.e. failure to fulfil academic obligations, and problematic health behaviours, such as substance use [6,7].*

*Given the high prevalence of mental disorders among university students, there has been a burgeoning interest in detecting potential protective skills that could be enhanced in order to improve their mental health, such as mindfulness, self-compassion, and psychological flexibility [8, 9].”*

•	The focus of the paper is on dispositional mindfulness, but the introduction only briefly mentions how mindfulness trainings are related to stress. The connection between dispositional mindfulness and stress, especially among college students, needs to be described to better set up the paper.

**Answer:** Following reviewer’s suggestion, we have described the connection between trait mindfulness and stress in university students (page 3):

*“Many studies have consistently indicated the negative association of mindfulness, compassion, and psychological flexibility with psychological distress in general populations [14,15]. Specifically in university students, for instance, Burger et al. [16] investigated the associations between psychological distress and mindfulness in a sample of 174 university students and found that higher levels of mindfulness (especially the facet of acting with awareness) significantly predicted lower levels of psychological distress. Similarly, Muro et al. [17] found that mindfulness was associated with higher levels of life satisfaction in a sample of Spanish university students, a result which is consistent with the multicentre cross-sectional study of Salvarani et al. [18], who found that undergraduate nursing students (n = 622) with higher dispositional mindfulness scores had lower levels of psychological distress.”*

•	Self-compassion is suggested as a potential mediator of the relationship between mindfulness and happiness. By bringing this up, I wondered if the authors would test this mediation with stress as an outcome. The focus of the paragraph should be about the association between self-compassion and stress among college students.

**Answer:** Following reviewer’s suggestion, we have focused the paragraph on the association between self-compassion and stress among college students (page 3):

*“Regarding compassion, Chan et al. [19] have found in a sample of 536 university students that compassion for others and self-compassion were linked to lower levels of psychological distress. In addition, Rahmandani et al. [20] found a significant negative association between self-compassion and several dimensions of distress (e.g. loss of confidence, anxiety), suggesting that high self-compassion might explain a lower level of distress in university students. Both compassion for others and self-compassion have consistently been found to be related to trait mindfulness (e.g. [21]).”*

•	Experiential avoidance is discussed as a potential moderator of several associations, including anxiety, addictions, etc. Again, the authors need to discuss literature about how it is related to stress in particular to better set up the study aims.

**Answer:** Following reviewer’s suggestion, we have described the connection between experiential avoidance and stress in university students (page 3-4):

*“Regarding psychological flexibility, previous studies have focused on experiential avoidance, which is a process aimed at trying to facilitate psychological flexibility. Experiential avoidance is defined as a “phenomenon that occurs when a person is unwilling to remain in contact with particular private experiences (e.g., bodily sensations, emotions, thoughts, memories, behavioural predispositions) and takes steps to alter the form or frequency of these events and the contexts that occasion them” ([22], p. 1154). For instance, Farr et al. [23] found that experiential avoidance was a significant predictor of psychological distress, this positive association being moderated by self-compassion. Specifically, these authors showed that individuals high in self-compassion reported lower levels of depressive symptoms across low to high experiential avoidance levels than individuals with low self-compassion levels [23]. In addition, Chou et al. [24] found in a sample of 500 university students that experiential avoidance was a significant predictor of Internet addiction, significant depression, and suicidality. Finally, experiential avoidance and self-compassion are strongly and negatively associated [25]. Similarly, mindfulness has been found to negatively correlate with experiential avoidance [26].”*

•	It will also be beneficial to discuss how mindfulness, self-compassion, and experiential avoidance are associated with each other.

**Answer:** Following reviewer’s suggestion, we have described the connection between these three variables (pages 3-4):

*“Both compassion for others and self-compassion have consistently been found to be positively related to trait mindfulness (e.g. [21]).”*

*[…]*

*“Finally, experiential avoidance and self-compassion are strongly and negatively associated [25]. Similarly, mindfulness has been found to negatively correlate with experiential avoidance [26].”*

*[…]*

*“Mindfulness, self-compassion, and experiential avoidance are three interrelated processes. According to Neff [27], there is a reciprocal association between mindfulness and self-compassion, in which each one is necessary for facilitating and enhancing the other. […]. Furthermore, being unjudgmental and acceptance (central facets of mindfulness) can be considered the beneficial counterparts of experiential avoidance [26]. Likewise, mindfulness and experiential avoidance are drawn from very different theoretical contexts, i.e. Eastern philosophy and functional contextualism, respectively.”*

•	The study aims should be reframed to focus on exploring associations among mindfulness, self-compassion, and experiential avoidance with stress. Do the authors have any hypotheses?

**Answer:** Following reviewer’s suggestion, we have reframed the study aims to focus on those associations. The hypotheses have been emphasized (page 5-6):

*“Therefore, the aim of the current study was to explore the relative contributions of three processes that are therapeutically relevant to perceived stress (i.e. trait mindfulness, self-compassion, and experiential avoidance), a critical construct among university students who are at risk of burnout, depression, and anxiety. According to the literature, we expected that mindfulness, self-compassion, and experiential avoidance would explain a significant proportion of the variance of perceived stress after controlling for relevant sociodemographic and academic variables such as age, gender, or having a scholarship. The direction of the relationship between mindfulness/self-compassion and perceived stress was expected to be negative (inverse). Alternatively, a positive relationship was expected between experiential avoidance and stress. No prior hypotheses were established on the strength of the associations due to the scarce number of previous studies.”*

Methods

•	Additional descriptions of study measures should be included to better interpret some of the measures. How was presence of family support measured (yes/no; good/bad)? How were overall scores created (summed, averaged)?

**Answer:** Following reviewer’s suggestion, we have included that information. Concretely, we have specified the response options for the sociodemographic and academic variables that were categorical (page 8):

*“The following sociodemographic characteristics were recorded using an ad hoc questionnaire (response options): age, gender (male/female), having a partner (yes/no), number of children, perceived presence of family support (insufficient/good/very good), employment (yes/no), and emancipation status (yes/no). Likewise, the following academic variables were recorded: having a scholarship (yes/no), which academic year the student was in, total study hours per week, and number of failed subjects over the previous examination period. Along with this survey, participants completed the aspects described below as part of a pencil-and-paper or online set of measurements.”*

Moreover, we have specified how total scores of the questionnaires were calculated (pages 9-10):

*“The Perceived Stress Questionnaire-Short Form (PSQ-SF; [36]) […] The total score ranges from 0 to 1 and can be calculated by adding all items, subtracting 24, and dividing by 72 [37].*

*[…]*

*The Five Facets Mindfulness Questionnaire-Short Form (FFMQ-SF; [38]) […] Two subscales (describing and non-reactivity to inner experience) demonstrated inadequate internal consistency (Cronbach’s α < .70), thus only the total score, which was computed by calculating the sum of all items, was retained for the analyses. Higher scores represent greater levels of mindfulness, with the total scores ranging from 20 to 100 points.*

*[…]*

*The Self-Compassion Scale-Short Form (SCS-SF; [39]) […] A total score can be computed by calculating the sum of all items after reversing those that are negatively worded (scores ranging from 12 to 60). Higher scores indicate more tendency towards self-compassion.*

*[…]*

*The Acceptance and Action Questionnaire-II (AAQ-II; [41]) […] A total score can be calculated by adding up all items, with scores ranging from 7 to 49. Higher scores indicate a higher tendency to present experiential avoidance.”*

•	Why did the authors choose to explore the five facets of mindfulness instead of an overall index score? This rationale should be included. The non-reactivity subscale demonstrated low reliability, which should be noted as a limitation. Was a Spanish version of the mindfulness scale used? If so, this should be stated.

**Answer:** We agree with the reviewer. Although we wanted to know the contribution of each of the five facets of mindfulness on perceived stress, not only the non-reactivity subscale, but also the describing subscale showed a low reliability. Thus, we have calculated a total score and used it in the analysis. It showed an adequate reliability. In addition, we have specified that we used the Spanish version of the instrument (page 9):

*“The Five Facets Mindfulness Questionnaire-Short Form (FFMQ-SF; [38]) is a 20-item questionnaire that measures the five facets of mindfulness: observing, describing, acting with awareness, non-judging of inner experience, and non-reactivity to inner experience. The response format is a 5-point scale (1 = “never or very rarely true”, 5 = “very often or always true”). Tran et al. [38] validated the German and the Spanish version of this FFMQ-SF. Two subscales (describing and non-reactivity to inner experience) demonstrated inadequate internal consistency (Cronbach’s α < .70), thus only the total score, which was computed by calculating the sum of all items, was retained for the analyses. Higher scores represent greater levels of mindfulness, with the total scores ranging from 20 to 100 points. In the current sample, the total score showed an adequate internal consistency with a Cronbach’s α value of 0.76.”*

*38.	Tran US, Cebolla A, Glück TM, Soler J, Garcia-Campayo J, Moy T von. The Serenity of the Meditating Mind: A Cross-Cultural Psychometric Study on a Two-Factor Higher Order Structure of Mindfulness, Its Effects, and Mechanisms Related to Mental Health among Experienced Meditators. PLOS ONE. 2014;9(10):e110192.*

Finally, the inadequate internal consistency of the FFMQ subscales has been mentioned as a limitation of the study (page 20):

*“These findings should be interpreted with caution regarding the following limitations. Firstly, our findings are based on self-reported measurements and could suffer from social desirability bias. Moreover, two subscales of the mindfulness measurement showed inadequate internal consistency, so only the total score could be used.”*

Procedure

•	What were the students informed that the study was about?

**Answer:** Following reviewer’s suggestion, we have provided that information (page 10):

*“In particular, it was explained to them that the aim of the study was to investigate the association between their levels of perceived stress, mindfulness, self-compassion, and experiential avoidance.”*

•	What year was the study conducted in?

**Answer:** Following reviewer’s suggestion, we have provided that information (page 11):

*“The survey responses were collected from March 2016 to May 2016.”*

•	Were the students compensated for participating?

**Answer:** Following reviewer’s suggestion, we have provided that information (page 11):

*“There was no payment for participating. Returning a blank survey was accepted without any associated punishment.”*

•	Were there any differences between students who completed the survey online vs. those who completed the survey using a pencil and paper?

**Answer:** Regretfully, we are not able to identify which subjects responded online and which ones via paper-and-pencil. This aspect was not registered. Then, we have highlighted it in the limitations section as well as results might have been biased by these two different answering procedures (page 21):

*“Fifthly, results might be biased due to the followed self-selection sampling procedure and the use of two measurement procedures (in-person and online). In that regard, we were not able to check for differences between subjects answering online and via pencil and paper because it was not registered.”*

Results

•	How were missing data handled?

**Answer:** Missing data were handled with the ‘Exclude cases pairwise’ option. This option excluded the cases only if they are missing the data required for the specific analysis. They will still be included in any of the analyses for which they have the necessary information. It is strongly recommended instead of the ‘exclude cases listwise’ option, which include cases in the analysis only if it has full data on all of the variables included in the analysis. This information has been included in the Data Analysis section (page 12): *“Pairwise deletion was the technique used to handle missing data.”*

•	The authors stated that stress and other variables corresponded to “a medium score range.” What exactly do the authors mean by this? Are there cut-offs for clinically meaningful levels of stress? Are there references for interpreting what these values mean?

**Answer:** Following reviewers’ suggestions, we have focused on the hierarchical multiple regression. We have removed complementary analyses from the main document, i.e., those that were not necessary to answer the research question. Thus, we have removed this part of the text.

•	The paragraph describing the description of the sample repeats all of the info in Table 1. Unless the authors want to describe clinically meaningful cut-off scores based on the means of the variables, I think this information can be removed from the text and only included in the table.

**Answer:** Following reviewer’s suggestion, we have removed in-text information that was shown in the tables. Now, the paragraph describing the description of the sample provides the following information (page 6):

*“Table 1 displays all the sociodemographic, academic, and psychological characteristics for the total sample. The study sample was composed of 589 university teaching students from the San Vicente Mártir Catholic University of Valencia (Spain), of whom 81.2% were female, and the mean age was 22.13 years old (SD = 3.90; range: 18-48). All participants were White. The inclusion criteria were: (1) being ≥18 years old, (2) being an undergraduate student in the field of education, and (3) being able to understand written Spanish.”*

•	I suggest that the authors do not use a stepwise regression for their analyses. A stronger approach would be a hierarchical multiple regression based on variables associated with stress at the univariable level (correlations). This would still examine whether mindfulness, self-compassion, and experiential avoidance are associated with stress above the effects of sociodemographic and academic variables.

**Answer:** We agree with the reviewer. Following reviewer’s suggestion, we have re-analyzed our data conducting a hierarchical multiple regression (enter method, not stepwise) and re-organized the results section according to that. As reviewer suggests, in the hierarchical multiple regression we enter our variables in steps or blocks in a predetermined order (not letting the computer decide, as would be the case for stepwise regression). In the first block, we forced sociodemographic and academic variables responding into the analysis. This has the effect of statistically controlling for these variables. In the second step we entered the other independent variables (mindfulness, self-compassion, and experiential avoidance) into the model as a block being removed the possible effect of sociodemographic and academic variables. This information has been included in pages 11-12:

*“A hierarchical multiple regression was carried out to assess the ability of FFMQ-SF (mindfulness), SCS-SF (self-compassion), and AAQ-II (experiential avoidance) measurements to explain PSQ-SF (perceived stress) scores after controlling for the influence of sociodemographic and academic variables. All categorical sociodemographic and academic variables were recoded as dummy variables as follows: gender (0 = male, 1 = female), having a partner (0 = no, 1 = yes), children (0 = no, 1 = yes), perceived presence of family support (0 = no = insufficient, 1 = yes = good/very good), employment (0 = no, 1 = yes), left home (0 = no, 1 = yes), having a scholarship (0 = no, 1 = yes), which academic year the student was in (each academic year recoded as dummy variable; 0 = no, 1 = yes). These categorical variables along with age, total study hours per week, and number of failed subjects over the previous examination period were entered at Step 1. Additionally, FFMQ-SF, SCS-SF, and AAQ-II scores were entered at Step 2. Pairwise deletion was the technique used to handle missing data. Semi-partial correlation coefficients (sr) were examined to get an indication of the unique contribution of each variable to the total R square. Specifically, if this coefficient is squared, the percentage of unique variance explained by an independent variable in the dependent variable is obtained [44]. Preliminary analyses were conducted to check the assumptions of multicollinearity, outliers, normality, linearity, and homoscedasticity (S1 and S2 Tables and S3 Fig.), with no major violations noted. Continuing along the path laid down by previous studies [45], the hierarchical multiple regression was re-tested including an ad hoc SCS-SF total score for which the mindfulness and overidentification scores were removed (S4 Table). This analysis was performed due to the partial overlap between trait mindfulness and self-compassion measurements (i.e. FFMQ-SF and SCS-SF, respectively; [28]).”*

Tables

Table 1

- The authors should include a note under the table explaining what the abbreviations for the measures correspond to.
- Please add the range of scores for each measure within your sample.

**Answer:** Fixed. We have explained the abbreviations in a foot note under the table. Following reviewer’s suggestion, we have included also the possible range of the scores. Please, see below the last part of Table 1 (page 8):

*“Table 1. Sociodemographic, Academic, and Psychological Characteristics of the Sample (n = 589)*

| *Psychological variables* | *M (SD)* | *Minimum* | *Maximum* |
| --- | --- | --- | --- |
| *PSQ-SF (0 - 1)* | *0.42 (0.17)* | *0.01* | *0.97* |
| *FFMQ-SF (20 - 100)* | *63.70 (9.52)* | *25* | *97* |
| *SCS-SF (12 - 60)* | *36.74 (8.51)* | *12* | *60* |
| *AAQ-II (7 - 49)* | *22.85 (9.11)* | *7* | *48* |

*Note. n = frequencies; % = percentages; M = mean; SD = standard deviation; PSQ-SF = Perceived Stress Questionnaire-Short Form; FFMQ-SF = Five Facets Mindfulness Questionnaire-Short Form; SCS-SF = Self-Compassion Scale-Short Form; AAQ-II = The Acceptance and Action Questionnaire-II. Range of possible scores are shown in brackets.”*

Table 2

- This table is very large and difficult to read.
- The key study variables should be listed at the top of the table (stress, mindfulness, self-compassion, and experiential avoidance).
- The authors should include a note under the table explaining how variables were dichotomized. For example, what does a negative vs. positive correlation for gender mean?

**Answer:** We agree with the reviewer in the inconvenience of the table size. Moreover, after reflecting about its importance in the study, we have decided to reduced it and include it as part of the *Supplementary Materials (S1 Table)*. It shows complementary results. Furthermore, for more clarity, we have improved its readability following reviewer’s suggestions (options for dichotomous variables and a note explaining the abbreviations were included). Finally, how variables were dichotomized is included in the Data Analysis section (page 11):

*“All categorical sociodemographic and academic variables were recoded as dummy variables as follows: gender (0 = male, 1 = female), having a partner (0 = no, 1 = yes), children (0 = no, 1 = yes), perceived presence of family support (0 = no = insufficient, 1 = yes = good/very good), employment (0 = no, 1 = yes), left home (0 = no, 1 = yes), having a scholarship (0 = no, 1 = yes), which academic year the student was in (each academic year recoded as dummy variable; 0 = no, 1 = yes).”*

Discussion

•	The first paragraph does not highlight the main findings from the regression.

**Answer:** Following reviewer’s suggestion, we have rewritten the first paragraph of the discussion section. Now it highlights the main findings from the regression (pages 16-17):

*“This study examined the explanatory power of mindfulness, self-compassion, and experiential avoidance with regards to perceived stress in a sample of university students while controlling for the influence of relevant sociodemographic and academic variables. When all of them were taken into consideration as potential explanatory variables of perceived stress in the regression model, mindfulness, self-compassion, and experiential avoidance explained a greater variance of perceived stress than all sociodemographic and academic variables together. In particular, experiential avoidance was by far the most explanatory variable of levels of perceived stress (direct association). Self-compassion was also a significant explanatory variable that was inversely associated with perceived stress, but to a lower degree. Finally, trait mindfulness showed marginal additive explanatory power in the regression, but when self-compassion scores did not account for mindfulness and overidentification items, mindfulness was shown as a significant explanatory variable inversely associated with perceived stress. Regarding sociodemographic and academic variables, high total study hours per week, having a partner (vs. being single), being female (vs. being male), and being older were significantly associated with high levels of perceived stress. In addition, perceived family support was found to be a protective variable, a good/very good perception of family support being associated with lower levels of perceived stress. Finally, having children, being in employment, having left the family home, having a scholarship, the academic year, and the number of failed subjects were not significant contributors to higher levels of perceived stress. Thus, perceived stress in our sample was positively associated with experiential avoidance, which could be regarded as a psychological variable with potential risk. In contrast, perceived stress was negatively correlated with self-compassion and mindfulness, which, in turn, could be seen as protective factors.”*

•	Some facets of mindfulness were not associated in the direction as other facets. Why do the authors think that is?

•	Why do the authors think that the acting with awareness facet in particular was important, relative to the other facets? What are the implications of these findings?

**Answer:** Finally, we only used the total score of the FFMQ-SF. Then, information regarding the facets was removed. It has been explained in the Instruments section (page 9):

*“Two subscales (describing and non-reactivity to inner experience) demonstrated inadequate internal consistency (Cronbach’s α < .70), thus only the total score, which was computed by calculating the sum of all items, was retained for the analyses.”*

Also, we acknowledged the lack of reliability of the FFMQ-SF facets as a limitation of the study (page 20):

*“Moreover, two subscales of the mindfulness measurement showed inadequate internal consistency, so only the total score could be used.”*

•	The authors mention how self-compassion overlaps with mindfulness. Indeed, self-compassion includes a component of mindfulness. What are the potential methodological concerns with using both constructs in the regression model? Did the authors test self-compassion with the mindfulness component removed?

**Answer:** The overlap between variables was not a methodological concern for our analysis, multicollinearity analysis (correlations among variables, tolerance and VIF coefficients shown in supplementary material) demonstrated significant but not large enough correlations among these independent variables to preclude regression as well as admissible tolerance and VIF values. The origin of the overlap relies on the theoretical base of the self-compassion measure. However, following reviewer’s suggestion we re-tested the multiple regression including self-compassion with the mindfulness and overidentification components removed. The complete output was included as *Supplementary Material (S4 table)* and results were commented in the main document. This procedure was explained in data analysis (page 12):

*“Continuing along the path laid down by previous studies [45], the hierarchical multiple regression was re-tested including an ad hoc SCS-SF total score for which the mindfulness and overidentification scores were removed (S4 Table). This analysis was performed due to the partial overlap between trait mindfulness and self-compassion measurements (i.e. FFMQ-SF and SCS-SF, respectively; [28]).”*

Here, the respective paragraph in the results section (page 16):

*“When the hierarchical multiple regression was re-tested with the SCS-SF total score calculated without adding the mindfulness and overidentification items scores (S4 Table), FFMQ-SF did significantly explain PSQ-SF scores (sr = -0.07; p = .015), along with the aforementioned variables. In particular, AAQ-II, SCS-SF, and FFMQ-SF scores uniquely explained around 11%, 2%, and 0.55%, respectively, of the variance in PSQ-SF scores.”*

Finally, the paragraph in the discussion in which we explain this result (pages 18-19):

*“Even though the inverse association between dispositional mindfulness and psychopathological symptoms has been consistently reported [52], our results showed a marginally significant relationship between mindfulness as a trait and perceived stress. One possible explanation for that unexpected result was the partial overlap between mindfulness (FFMQ) and self-compassion (SCS) measurements [49]. That is, Neff’s model [27] includes mindfulness (vs. overidentification) as one of the three positive components of self-compassion, which is mirrored in the SCS. In fact, when the overlap between these measurements was solved, mindfulness and perceived stress association were found to be statistically significant. Therefore, the marginal association that was initially found was mostly related to psychometric issues that future research should address using other compassion measurements such as the Sussex-Oxford Compassion Scales [21].”*

•	Having an in-person, pencil and paper survey could cause potential bias in responses. This should be discussed.

**Answer**: Following reviewer’s suggestion, the data recollection issue has been included as a limitation of the study (pages 21):

*“Fifthly, results might be biased due to the followed self-selection sampling procedure and the use of two measurement procedures (in-person and online). In that regard, we were not able to check for differences between subjects answering online and via pencil and paper because it was not registered.”*

Other

•	Sometimes abbreviations for stress “PSQ” are used and other times it is referred to as “stress”. Be more consistent throughout the text to reduce confusion.

•	Sometimes the variables are referred to as “academic” variables and other time “occupational” variables (table titles; section headings). Be more consistent throughout the text to reduce confusion.

**Answer:** Following reviewer’s suggestion, we have used more consistently stress abbreviations throughout the paper and the term academic variables has been used, avoiding variants and synonyms. Concretely, we referred to stress as PSQ in the data analysis and results sections, in the rest of the document we said perceived stress.

•	There are several grammatical and spelling errors.

**Answer:** Following reviewer’s suggestion, the revised version of the manuscript has been proofread by a native English speaker with experience in science writing.

•	Have the data from this study been reported elsewhere?

**Answer:** Data from this study has been uploaded in OSF: https://osf.io/g2sfp/.

**Reviewer #2**

The manuscript reports findings from a cross-sectional survey study of university students in education regarding perceived stress and therapeutically relevant processes. The manuscript is well-written and generally clear. Overarching concerns include: 1) a lack of unifying theme among the processes examined or a framework to unite them therapeutically (which will help with clinical implications) and 2) the potential for bias in the statistical approach that was selected.

**Authors**: We would like to thank you very much for your kind and thoughtful comments. We will provide an answer after each comment below.

Abstract

The authors note that there is a high “prevalence” of perceived stress among college students. Please revise to indicate high levels, as prevalence has another meaning which the authors did not measure.

**Answer**: Following reviewer’s suggestion, we have modified that sentence (page 1):

*“University students constitute a population that is highly vulnerable to developing mental health problems, such as distress.”*

Introduction

Authors should define the “third wave” and “fourth wave” therapies for readers who may be unfamiliar with what approaches fall within each wave.

**Answer**: Following reviewer’s suggestion, we have briefly explained the core feature of third wave therapies. Also, we have removed the mention to fourth wave therapies in order to simplify the introduction section (page 2):

*“Given the high prevalence of mental disorders among university students, there has been a burgeoning interest in detecting potential protective skills that could be enhanced in order to improve their mental health, such as mindfulness, self-compassion, and psychological flexibility [8, 9]. These are three psychological constructs of “third wave” cognitive behavioural therapy, in which the focus is on the relationship between the individual and his/her thoughts and emotions [10].”*

Experiential avoidance is defined as the opposite of psychological flexibility, though there is quite a bit of debate about this in the ACT literature. The authors might just define experiential avoidance, mention that this is a process addressed in trying to facilitate psychological flexibility, or note it as a part of psychological inflexibility (which is thought of as the opposite of psychological flexibility in some of the recent literature).

**Answer**: Following reviewer’s suggestion, we have added the original definition of experiential avoidance and included the reviewer’s idea (page 3):

*“Regarding psychological flexibility, previous studies have focused on experiential avoidance, which is a process aimed at trying to facilitate psychological flexibility. Experiential avoidance is defined as a “phenomenon that occurs when a person is unwilling to remain in contact with particular private experiences (e.g., bodily sensations, emotions, thoughts, memories, behavioural predispositions) and takes steps to alter the form or frequency of these events and the contexts that occasion them” ([22], p. 1154).”*

A clearer picture of the processes being examined fit together may be helpful through a unifying theme before each is defined individually. For example, the non-judgment process of mindfulness often includes elements of self-compassion. The act with awareness and nonreactivity facets of mindfulness often include some form of acceptance (or lack of avoidance). A unifying theme would help to illustrate how these are all interrelated processes and justify the later analytic choice.

**Answer**: Following reviewer’s suggestion, we have added a paragraph in which we describe how these processes interrelate (pages 3-4):

*“**Both compassion for others and self-compassion have consistently been found to be positively related to trait mindfulness (e.g. [21]). […]* *Finally, experiential avoidance and self-compassion are strongly and negatively associated [25]. Similarly, mindfulness has been found to negatively correlate with experiential avoidance [26].*

*Mindfulness, self-compassion, and experiential avoidance are three interrelated processes. According to Neff [27], there is a reciprocal association between mindfulness and self-compassion, in which each one is necessary for facilitating and enhancing the other. For example, a self-compassionate response requires a mindful approach to painful thoughts and feelings. However, an important distinction between them is that mindfulness is applied to pleasant, neutral, or unpleasant experiences, whereas self-compassion is focused on suffering. Moreover, self-compassion is focused on the global self, meanwhile mindfulness can be applied to the self as well as to thoughts, feelings, and sensations [28**]. Furthermore, being unjudgmental and acceptance (central facets of mindfulness) can be considered the beneficial counterparts of experiential avoidance [26]. Likewise, mindfulness and experiential avoidance are drawn from very different theoretical contexts, i.e. Eastern philosophy and functional contextualism, respectively.”*

The aims/objective statement at the end of the introduction could be more clearly phrased. The authors wanted to explore the relative contributions of three therapeutically relevant processes to perceived stress, a critical construct among college students who are studying for a career in a field known to be at risk for burnout, depression, and anxiety. The authors should emphasize their question and its importance here.

**Answer:** Following reviewer’s suggestions, we have rephrased the objective statement and modified the whole paragraph (pages 5-6):

*“Therefore, the aim of the current study was to explore the relative contributions of three processes that are therapeutically relevant to perceived stress (i.e. trait mindfulness, self-compassion, and experiential avoidance), a critical construct among university students who are at risk of burnout, depression, and anxiety. According to the literature, we expected that mindfulness, self-compassion, and experiential avoidance would explain a significant proportion of the variance of perceived stress after controlling for relevant sociodemographic and academic variables such as age, gender, or having a scholarship. The direction of the relationship between mindfulness/self-compassion and perceived stress was expected to be negative (inverse). Alternatively, a positive relationship was expected between experiential avoidance and stress. No prior hypotheses were established on the strength of the associations due to the scarce number of previous studies.”*

Method

Were participants compensated for participating, or did they get course credit?

**Answer:** Following reviewer’s suggestion, we have specified that participants were not compensated for participating. Likewise, as mentioned, no participation was accepted without any associated punishment (page 11):

*“The survey response time was approximately 20 minutes. There was no payment for participating. Returning a blank survey was accepted without any associated punishment.”*

For the PSQ measure, the authors could provide more information about the original validation study, including construct, convergent, discriminant, or incremental validity. Thank you for noting that your group validated this for use among Spanish university students, and great work – it’s great to see follow-up validation studies being done. The authors later report a category of medium score of stress in the Results – the way this score is derived should be reported in the section regarding this measure.

**Answer:** Following reviewer’s suggestion, we have improved the description of the PSQ as follows. Also, we have removed that part of the results section. Following previous reviewers’ suggestions, we removed complementary analysis and focused on the main analysis of the paper, i.e., the hierarchical multiple regression, which answers the research question (page 9):

*“The Perceived Stress Questionnaire-Short Form (PSQ-SF; [36]) is a 24-item scale that assesses perceived stress during the previous 30 days. The response format is a 4-point scale (from 1 = “almost never” to 4 = “almost always”). This questionnaire was validated for Spanish university students and showed adequate internal consistency and construct validity [37]. The total score ranges from 0 to 1 and can be calculated by adding all items, subtracting 24, and dividing by 72 [37]. Higher scores represent greater levels of perceived stress. The PSQ has demonstrated excellent internal consistency in the present sample with a Cronbach’s α value of 0.93.”*

There is some confusion with the FFMQ-SF scale. You note that it has 20 items with 5 items for 5 subscales, but this results in 25 items. The citation is also for the original scale, not the short form. The authors might also address the low internal consistency of the observing, describing, and non-reactivity subscales. Was the internal consistency for the total score improved?

**Answer:** Following reviewer’s suggestion, we have improved the description of this measure and addressed the problem of internal consistency of the FFMQ subscales. As you can see below, only the total score was calculated because the poor reliability of some facets (page 9):

*“The Five Facets Mindfulness Questionnaire-Short Form (FFMQ-SF; [38]) is a 20-item questionnaire that measures the five facets of mindfulness: observing, describing, acting with awareness, non-judging of inner experience, and non-reactivity to inner experience. The response format is a 5-point scale (1 = “never or very rarely true”, 5 = “very often or always true”). Tran et al. [38] validated the German and the Spanish version of this FFMQ-SF. Two subscales (describing and non-reactivity to inner experience) demonstrated inadequate internal consistency (Cronbach’s α < .70), thus only the total score, which was computed by calculating the sum of all items, was retained for the analyses. Higher scores represent greater levels of mindfulness, with the total scores ranging from 20 to 100 points. In the current sample, the total score showed an adequate internal consistency with a Cronbach’s α value of 0.76.”*

Regarding the SCS-SF, the authors did not use the subscales as they did for the FFMQ-SF. Can the authors justify this choice? One alternative would be to use total scores for all scales, and if significant associations are uncovered in the regression, conduct an exploratory analysis with the subscales instead of the total score to identify the most important processes.

**Answer:** Following reviewer’s suggestion, we have used the total scores of all measures. You can find below which variables were included in the regression (page 11):

*“A hierarchical multiple regression was carried out to assess the ability of FFMQ-SF (mindfulness), SCS-SF (self-compassion), and AAQ-II (experiential avoidance) measurements to explain PSQ-SF (perceived stress) scores after controlling for the influence of sociodemographic and academic variables.”*

The authors specify March to May completion dates, but they do not specify the year. Given the implications of the COVID-19 pandemic for university students, specify the year if possible, and if not, whether the data were collected pre- or peri-pandemic.

**Answer:** Following reviewer’s suggestion, we have included this information (page 11):

*“The survey responses were collected from March 2016 to May 2016.”*

The authors specify prior studies as justification to recode sociodemographic and academic variables, but they might offer a statistical justification instead. At the very least, as a reviewer, I cannot judge the appropriateness of this approach without breaking the masking of the review process since authors were not specified.

**Answer:** Following editor’s and reviewer’s suggestion, we have unblinded the manuscript, so now all references can be checked. The recodification of categorial sociodemographic and academic variables have been better explained (page 11):

*“All categorical sociodemographic and academic variables were recoded as dummy variables as follows: gender (0 = male, 1 = female), having a partner (0 = no, 1 = yes), children (0 = no, 1 = yes), perceived presence of family support (0 = no = insufficient, 1 = yes = good/very good), employment (0 = no, 1 = yes), left home (0 = no, 1 = yes), having a scholarship (0 = no, 1 = yes), which academic year the student was in (each academic year recoded as dummy variable; 0 = no, 1 = yes).”*

The authors might consider not using a stepwise approach to the regression, as it generally is thought to bias R2 values to be higher, deflate standard errors, and narrow confidence intervals. In addition, this exacerbates collinearity problems, which is likely to be the biggest issue with stepwise and the authors’ data, as the independent variables are at least modestly correlated. The authors should consider hierarchical regression where all variables originally included are reported in the final models. Below are a few citations to consider about stepwise. If the authors choose to keep the stepwise approach, these limitations should be acknowledged thoroughly and justification of this approach should be provided.

Thompson, B. (1995). Stepwise regression and stepwise discriminant analysis need not apply here: A guidelines editorial. Educational and psychological measurement, 55(4), 525-534.

Henderson, D. A., & Denison, D. R. (1989). Stepwise regression in social and psychological research. Psychological Reports, 64(1), 251-257.

Whittingham, M. J., Stephens, P. A., Bradbury, R. B., & Freckleton, R. P. (2006). Why do we still use stepwise modelling in ecology and behaviour?. Journal of animal ecology, 75(5), 1182-1189.

**Answer:** We agree with the reviewer’s comment, so we have computed a hierarchical multiple regression analysis (pages 11-12):

*“A hierarchical multiple regression was carried out to assess the ability of FFMQ-SF (mindfulness), SCS-SF (self-compassion), and AAQ-II (experiential avoidance) measurements to explain PSQ-SF (perceived stress) scores after controlling for the influence of sociodemographic and academic variables. All categorical sociodemographic and academic variables were recoded as dummy variables as follows: gender (0 = male, 1 = female), having a partner (0 = no, 1 = yes), children (0 = no, 1 = yes), perceived presence of family support (0 = no = insufficient, 1 = yes = good/very good), employment (0 = no, 1 = yes), left home (0 = no, 1 = yes), having a scholarship (0 = no, 1 = yes), which academic year the student was in (each academic year recoded as dummy variable; 0 = no, 1 = yes). These categorical variables along with age, total study hours per week, and number of failed subjects over the previous examination period were entered at Step 1. Additionally, FFMQ-SF, SCS-SF, and AAQ-II scores were entered at Step 2. Pairwise deletion was the technique used to handle missing data. Semi-partial correlation coefficients (sr) were examined to get an indication of the unique contribution of each variable to the total R square. Specifically, if this coefficient is squared, the percentage of unique variance explained by an independent variable in the dependent variable is obtained [44]. Preliminary analyses were conducted to check the assumptions of multicollinearity, outliers, normality, linearity, and homoscedasticity (S1 and S2 Tables and S3 Fig.), with no major violations noted. Continuing along the path laid down by previous studies [45], the hierarchical multiple regression was re-tested including an ad hoc SCS-SF total score for which the mindfulness and overidentification scores were removed (S4 Table). This analysis was performed due to the partial overlap between trait mindfulness and self-compassion measurements (i.e. FFMQ-SF and SCS-SF, respectively; [28]).”*

Results

What is the variable “number of failed subjects”? The authors might explain this further in the method, as this is not entirely clear when reported in the results/table.

**Answer**: The variable “number of failed subjects” means the number of failed subjects over the previous examination period. Following reviewer’s suggestion, the description of the sociodemographic and academic variables has been updated as follows (page 8):

*“The following sociodemographic characteristics were recorded using an ad hoc questionnaire (response options): age, gender (male/female), having a partner (yes/no), number of children, perceived presence of family support (insufficient/good/very good), employment (yes/no), and emancipation status (yes/no). Likewise, the following academic variables were recorded: having a scholarship (yes/no), which academic year the student was in, total study hours per week, and number of failed subjects over the previous examination period. Along with this survey, participants completed the aspects described below as part of a pencil-and-paper or online set of measurements.”*

The interrelations among the processes should be specified in-text as these bear on the potential for multicollinearity in the regression analyses. I’m not sure how to solve this, as large correlation matrices are hard to manage, but it is very challenging to interpret a correlation matrix that stretches across three pages.

**Answer**: Following reviewers’ considerations, we have decided to include this table as supplementary material. As well, it has been simplified. Regarding multicollinearity and other multiple regression assumptions, information provided as supplementary material shows tolerable correlations, tolerance, and VIF values (page 12):

*“Preliminary analyses were conducted to check the assumptions of multicollinearity, outliers, normality, linearity, and homoscedasticity (S1 and S2 Tables and S3 Fig.), with no major violations noted.”*

Discussion

The discussion section speaks to the findings and contextualizes them in the broader literature, but this section could benefit from the unifying theme that I suggest in the introduction section comments. This would help to provide readers a clear next step with regard to clinical implications and future research. These processes are all part of Acceptance and Commitment Therapy or more broadly, mindfulness- and acceptance-based therapies, and if discussed as part of these approaches, it will clarify how they all interrelate. The authors might also provide context around the known risk of educators for burnout, anxiety, depression, and other forms of distress-related disorders. These findings could be use to support prevention efforts during education that will help educators have better occupational health once on the job.

**Answer**: Following reviewer’s suggestion, we have emphasized the potential usefulness of our results to inform intervention in university students. These important issues have been discussed in the following manner:

(Page 17) *“Thus, perceived stress in our sample was positively associated with experiential avoidance, which could be regarded as a psychological variable with potential risk. In contrast, perceived stress was negatively correlated with self-compassion and mindfulness, which, in turn, could be seen as protective factors.”*

(Page 19) *“Up to now, mindfulness-based interventions for university students have shown promising effectiveness [8,57], though our results suggest that comprehensive programmes more oriented at fostering other crucial process variables for stress reduction such as experiential avoidance and self-compassion are also needed in students showing greater levels of perceived stress. Therefore, these variables should be considered as targets in programmes aimed at fostering wellbeing and mental health in university students. Programmes aimed at increasing certain psychological skills such as Acceptance and Commitment Therapy (ACT; [58,59]) or the Mindful self-compassion programme [60] should be considered when choosing interventions for this population.”*

(Pages 21-22): *“Thus, our findings underpin the need for formative interventions that would aim firstly to reduce experiential avoidance levels, secondly to promote self-compassion, and thirdly to use mindfulness training as a psychotherapeutic aid. Adequate efforts should be made by higher education institutions to recognise and address university students’ mental health challenges and towards facilitating their mental health recovery, by reducing the long-term consequences of the pandemic on mental health. Thus, these three variables that can be trained and modified could be included as key factors in specific psychological interventions targeted at university students, as previous studies already suggested.”*

Finally, please, excuse our modesty when informing about the clinical and practical implications of our study. In fact, it is our view that no single study can be used to inform clinical, policy, prevention, or practice implications. Each study provides suggestive evidence, but whether the findings of the study can be replicated is questionable, especially given the limitations of the study. Thus, the findings of no one study should be used to tell readers what they ought to be doing based on that study. The point is, how can one study with methodological limitations provide directions for clinical work or practice when there is a whole branch of psychology dealing with evidence-based research to inform treatments?

Concerns about the AAQ-II and its modest correlation with negative affect should be acknowledged. This could be the reason for its prediction of perceived stress, and it is a limitation of the measure (not your study).

Tyndall, I., Waldeck, D., Pancani, L., Whelan, R., Roche, B., & Dawson, D. L. (2019). The Acceptance and Action Questionnaire-II (AAQ-II) as a measure of experiential avoidance: Concerns over discriminant validity. Journal of Contextual Behavioral Science, 12, 278-284.

**Answer**: Following reviewer’s suggestion we have included that aspect about the AAQ-II as a limitation of the study (page 20): *“These findings should be interpreted with caution regarding the following limitations. Firstly, our findings are based on self-reported measurements and could suffer from social desirability bias. Moreover, two subscales of the mindfulness measurement showed inadequate internal consistency, so only the total score could be used. Secondly, the SCS and AAQ-II have been extensively criticised for being overly saturated with personality traits or distress rather than specifically measuring self-compassion and experiential avoidance, respectively [62-65], a phenomenon that suggests an inflated relationship with perceived stress in the present study.”*

In addition, the method for recruitment and whether compensation was provided should be included. If people self-selected into the study, this should be noted as a limitation.

**Answer**: Following reviewer’s suggestion, we have included that information in the methods section (page 11): *“The survey response time was approximately 20 minutes. There was no payment for participating. Returning a blank survey was accepted without any associated punishment.”*; also in the limitations section (page 21): *“Fifthly, results might be biased due to the followed self-selection sampling procedure and the use of two measurement procedures (in-person and online).”*

The authors report change in adjusted R2 and use this as the metric for defining which variables had the most explanatory power. I am curious how much the psychological processes contributed as a group as well, over and above the sociodemographic and academic variables. A hierarchical linear regression without the stepwise approach (with sociodemographic and academic variables in step 1, and the psychological variables in step 2) could answer this question and would important given their interrelations.

**Answer**: Following reviewer’s suggestion we have computed a hierarchical multiple regression analysis. In addition to R square, we interpreted semipartial correlation coefficients, which give us information about the explanatory contribution of each variable when the others in the model are controlled for (pages 11-12):

*“A hierarchical multiple regression was carried out to assess the ability of FFMQ-SF (mindfulness), SCS-SF (self-compassion), and AAQ-II (experiential avoidance) measurements to explain PSQ-SF (perceived stress) scores after controlling for the influence of sociodemographic and academic variables. All categorical sociodemographic and academic variables were recoded as dummy variables as follows: gender (0 = male, 1 = female), having a partner (0 = no, 1 = yes), children (0 = no, 1 = yes), perceived presence of family support (0 = no = insufficient, 1 = yes = good/very good), employment (0 = no, 1 = yes), left home (0 = no, 1 = yes), having a scholarship (0 = no, 1 = yes), which academic year the student was in (each academic year recoded as dummy variable; 0 = no, 1 = yes). These categorical variables along with age, total study hours per week, and number of failed subjects over the previous examination period were entered at Step 1. Additionally, FFMQ-SF, SCS-SF, and AAQ-II scores were entered at Step 2. Pairwise deletion was the technique used to handle missing data. Semi-partial correlation coefficients (sr) were examined to get an indication of the unique contribution of each variable to the total R square. Specifically, if this coefficient is squared, the percentage of unique variance explained by an independent variable in the dependent variable is obtained [44]. Preliminary analyses were conducted to check the assumptions of multicollinearity, outliers, normality, linearity, and homoscedasticity (S1 and S2 Tables and S3 Fig.), with no major violations noted. Continuing along the path laid down by previous studies [45], the hierarchical multiple regression was re-tested including an ad hoc SCS-SF total score for which the mindfulness and overidentification scores were removed (S4 Table). This analysis was performed due to the partial overlap between trait mindfulness and self-compassion measurements (i.e. FFMQ-SF and SCS-SF, respectively; [28]).”*

**Reviewer #3**

**Authors**: We would like to thank you very much for your kind and thoughtful comments. We will provide an answer after each comment below.

This paper explores the association between potential protective factors of health and perceived stress in university students. Given the high levels of stress in this group, I believe this paper is timely and needed. The paper is well written, and analyses are well conducted. My main concern is related to the use of the AAQ-II (Acceptance and Action Questionnaire), which has extensively been reported as overly saturated with personality traits or distress rather than specifically measuring experiential avoidance (Wolgast et al., 2014). I encourage the authors to include this a major limitation in the discussion section and to soften and/or adapt some of the implications they are making in the discussion.

**Answer**: Following reviewer’s suggestion we have included that aspect about the AAQ-II as a limitation of the study (page 20): *“Secondly, the SCS and AAQ-II have been extensively criticised for being overly saturated with personality traits or distress rather than specifically measuring self-compassion and experiential avoidance, respectively [62-65], a phenomenon that suggests an inflated relationship with perceived stress in the present study.”*

Please find below some additional minor suggestions:

I would recommend that the authors add a paragraph to the introduction explaining the broader rationale and context for 3rd wave interventions in this specific population.

**Answer**: Following reviewer’s suggestion we have emphasized the importance of mindfulness, compassion, and psychological flexibility constructs in the studied population. See below the new text (pages 2-4):

*“Given the high prevalence of mental disorders among university students, there has been a burgeoning interest in detecting potential protective skills that could be enhanced in order to improve their mental health, such as mindfulness, self-compassion, and psychological flexibility [8, 9]. These are three psychological constructs of “third wave” cognitive behavioural therapy, in which the focus is on the relationship between the individual and his/her thoughts and emotions [10]. Firstly, mindfulness can be defined as a two-component mood that involves the self-regulation of attention, i.e. paying attention to the immediate experience (including internal experiences such as thoughts), and a curious, open, and accepting orientation towards it [11]. Secondly, compassion is a cognitive, affective, and behavioural process that involves recognising suffering, common humanity, empathy, tolerance for uncomfortable feelings, and motivation to act or acting to alleviate the suffering of others and/or oneself [12]. Finally, psychological flexibility is defined as the ability to consciously make contact with the present moment and behave in a way that serves valued goals [13].*

*Many studies have consistently indicated the negative association of mindfulness, compassion, and psychological flexibility with psychological distress in general populations [14,15]. Specifically in university students, for instance, Burger et al. [16] investigated the associations between psychological distress and mindfulness in a sample of 174 university students and found that higher levels of mindfulness (especially the facet of acting with awareness) significantly predicted lower levels of psychological distress. Similarly, Muro et al. [17] found that mindfulness was associated with higher levels of life satisfaction in a sample of Spanish university students, a result which is consistent with the multicentre cross-sectional study of Salvarani et al. [18], who found that undergraduate nursing students (n = 622) with higher dispositional mindfulness scores had lower levels of psychological distress.*

*Regarding compassion, Chan et al. [19] have found in a sample of 536 university students that compassion for others and self-compassion were linked to lower levels of psychological distress. In addition, Rahmandani et al. [20] found a significant negative association between self-compassion and several dimensions of distress (e.g. loss of confidence, anxiety), suggesting that high self-compassion might explain a lower level of distress in university students. Both compassion for others and self-compassion have consistently been found to be positively related to trait mindfulness (e.g. [21]).*

*Regarding psychological flexibility, previous studies have focused on experiential avoidance, which is a process aimed at trying to facilitate psychological flexibility. Experiential avoidance is defined as a “phenomenon that occurs when a person is unwilling to remain in contact with particular private experiences (e.g., bodily sensations, emotions, thoughts, memories, behavioural predispositions) and takes steps to alter the form or frequency of these events and the contexts that occasion them” ([22], p. 1154). For instance, Farr et al. [23] found that experiential avoidance was a significant predictor of psychological distress, this positive association being moderated by self-compassion. Specifically, these authors showed that individuals high in self-compassion reported lower levels of depressive symptoms across low to high experiential avoidance levels than individuals with low self-compassion levels [23]. In addition, Chou et al. [24] found in a sample of 500 university students that experiential avoidance was a significant predictor of Internet addiction, significant depression, and suicidality. Finally, experiential avoidance and self-compassion are strongly and negatively associated [25]. Similarly, mindfulness has been found to negatively correlate with experiential avoidance [26].”*

More emphasis needs to be placed on the clinical implications of the findings. The discussion mentions what components should be included when designing an intervention for this group however, the authors do not consider or discuss factors that might not make these interventions feasible. Rather, the authors should consider adjustments that can be made through the university to make uptake of an intervention more likely.

**Answer**: Following reviewer’s suggestion we have improved the discussion section. Concretely, we have added suggestions for improving intervention in university students taking into account our results:

(Page 17) *“Thus, perceived stress in our sample was positively associated with experiential avoidance, which could be regarded as a psychological variable with potential risk. In contrast, perceived stress was negatively correlated with self-compassion and mindfulness, which, in turn, could be seen as protective factors.”*

(Page 19) *“Up to now, mindfulness-based interventions for university students have shown promising effectiveness [8,57], though our results suggest that comprehensive programmes more oriented at fostering other crucial process variables for stress reduction such as experiential avoidance and self-compassion are also needed in students showing greater levels of perceived stress. Therefore, these variables should be considered as targets in programmes aimed at fostering wellbeing and mental health in university students. Programmes aimed at increasing certain psychological skills such as Acceptance and Commitment Therapy (ACT; [58,59]) or the Mindful self-compassion programme [60] should be considered when choosing interventions for this population.”*

(Pages 21-22): *“Thus, our findings underpin the need for formative interventions that would aim firstly to reduce experiential avoidance levels, secondly to promote self-compassion, and thirdly to use mindfulness training as a psychotherapeutic aid. Adequate efforts should be made by higher education institutions to recognise and address university students’ mental health challenges and towards facilitating their mental health recovery, by reducing the long-term consequences of the pandemic on mental health. Thus, these three variables that can be trained and modified could be included as key factors in specific psychological interventions targeted at university students, as previous studies already suggested.”*

Please, excuse our modesty when informing about the clinical and practical implications of our study. In fact, it is our view that no single study can be used to inform clinical, policy, prevention, or practice implications. Each study provides suggestive evidence, but whether the findings of the study can be replicated is questionable, especially given the limitations of the study. Thus, the findings of no one study should be used to tell readers what they ought to be doing based on that study. The point is, how can one study with methodological limitations provide directions for clinical work or practice when there is a whole branch of psychology dealing with evidence-based research to inform treatments?

Issues with data collection that need better context:

• Ethnicity of participants not reported – there can be cultural differences influencing certain variables.

**Answer:** All participants were White; this detail has been included in the participants section (page 6): *“The study sample was composed of 589 university teaching students from the San Vicente Mártir Catholic University of Valencia (Spain), of whom 81.2% were female, and the mean age was 22.13 years old (SD = 3.90; range: 18-48). All participants were White.”*

• Data was collected on how much family support participants had. How exactly was this measured as it is likely that this was entirely subjective.

**Answer:** It refers to perceived family support; this detail has been included in the instruments section (page 8):

*“The following sociodemographic characteristics were recorded using an ad hoc questionnaire (response options): age, gender (male/female), having a partner (yes/no), number of children,* ***perceived presence of family support (insufficient/good/very good)****, employment (yes/no), and emancipation status (yes/no). Likewise, the following academic variables were recorded: having a scholarship (yes/no), which academic year the student was in, total study hours per week, and number of failed subjects over the previous examination period. Along with this survey, participants completed the aspects described below as part of a pencil-and-paper or online set of measurements.”*

The following limitation was added too (page 21):

*“Fourthly, an ad hoc variable was created to evaluate subjective family support, and further studies should also explore this construct with validated questionnaires.”*

• Previous clinical and medical history of participants not reported and nor has this been addressed.

• Authors do not address that data was collected at one time point only, this time point was most likely when students have exams and deadlines which would have influenced findings.

**Answer:** Following reviewer’s suggestion, those aspects have been included as limitations (page 20-21):

*“In this regard, several variables that may have had an influence on the relationship between the outcomes were not assessed. For instance, the role of the previous clinical and medical history of participants or personality traits on perceived stress are not computed. Another example is that data was collected at only one point in time, when students have exams and deadlines which may have influenced findings.”*
